# Association between changes in conditioned pain modulation efficiency and pain sensitivity: a randomized controlled trial

**DOI:** 10.3389/fpain.2026.1802533

**Published:** 2026-03-26

**Authors:** Priyanka Rana, Michael E. Robinson, Meryl J. Alappattu, Joseph Riley, Donovan J. Lott, Mark D. Bishop

**Affiliations:** 1 Department of Physical Therapy and Movement Science, University of Texas at El Paso, El Paso, TX, United States; 2 Department of Clinical and Health Psychology, University of Florida, Gainesville, FL, United States; 3 Center for Pain Rehabilitation Research, University of Florida, Gainesville, FL, United States; 4 Department of Physical Therapy, University of Florida, Gainesville, FL, United States; 5 Pain Research and Intervention Center of Excellence, University of Florida, Gainesville, FL, United States; 6 Department of Community Dentistry and Behavioral Science, University of Florida, Gainesville, FL, United States

**Keywords:** endogenous analgesia, neuroplasticity, pain modulation, quantitative sensory testing, thermal pain

## Abstract

Conditioned pain modulation (CPM) reflects endogenous inhibitory capacity and may demonstrate neuroplastic adaptations with repeated activation. However, the association between CPM efficiency changes and pain sensitivity remains unclear. This planned secondary analysis examined whether improvements in CPM efficiency were associated with changes in quantitative sensory testing (QST) measures and psychological factors in healthy adults. Study design, participants, and primary intervention effects have been reported previously in the primary trial publication. Sixty participants (aged 18–75 years) were randomized to high CPM exposure (five sessions), low CPM exposure (two sessions), or no CPM exposure groups. Multiple linear regression examined associations between changes in CPM efficiency and QST measures (thermal and pressure pain thresholds, tolerance, and ratings) and psychological factors (depression, anxiety, fear of pain, affect, and expectations), controlling for group and age. Improvements in CPM efficiency significantly predicted increases in heat pain threshold temperature (*β* = −1.90, *p* < 0.001, *R*^2^ = 0.43) and heat tolerance temperature (*β* = −0.56, *p* = 0.010, *R*^2^ = 0.34), indicating that participants required higher temperatures to detect and tolerate pain. However, pain intensity ratings at these thresholds remained unchanged. Age independently predicted smaller threshold improvements (*β* = −0.11, *p* < 0.001). No associations emerged between CPM changes and pressure pain measures, aftersensations, or psychological factors. CPM-induced neuroplasticity selectively enhanced thermal nociceptive detection through descending modulation without altering suprathreshold pain intensity encoding or affecting mechanical pain pathways. CPM induced with thermal stimuli functions as a thermal-specific biomarker rather than a global pain sensitivity indicator, with implications for clinical assessment and interventions targeting descending inhibitory pathways.

## 1 Introduction

Pain is a complex, multidimensional experience that emerges from dynamic interactions between nociceptive input, central nervous system processing, and psychosocial factors. Understanding the mechanisms underlying individual differences in pain perception and modulation represents a critical challenge in contemporary pain science, with significant implications for clinical practice and patient outcomes. Quantitative sensory testing (QST) provides an experimental framework for investigating these mechanisms, offering insights into both peripheral and central pain processing (2).

Conditioned pain modulation (CPM) represents a widely studied QST paradigm, providing an indirect behavioral assessment of the body's endogenous pain inhibitory capacity. While CPM is not currently established as a clinical diagnostic tool, research has demonstrated associations between CPM efficiency and chronic pain conditions, as well as treatment response (3–5), suggesting potential prognostic value warranting further investigation. CPM protocols evaluate how a noxious conditioning stimulus influences the perception of a spatially distant test stimulus, reflecting the function of diffuse noxious inhibitory control mechanisms (6–8). CPM efficiency represents an indirect indicator of the functional status of descending pain modulation systems that arise from higher brain regions, especially the periaqueductal gray (PAG) and rostral ventromedial medulla (RVM), and extend to the dorsal horn of the spinal cord where they regulate nociceptive signaling (9, 10).

Meta-analytic evidence suggests that individuals with chronic pain conditions generally demonstrate reduced CPM responses compared with pain-free controls (11), although considerable heterogeneity exists both within and between chronic pain populations and healthy individuals. This pattern suggests that deficient endogenous inhibition may contribute to pain chronification. CPM improvements have been linked to favorable treatment outcomes (3, 5), positioning CPM as a potentially valuable predictive biomarker (4, 6, 8).

Despite the clinical importance of CPM, longitudinal investigations examining how CPM efficiency changes relate to concurrent pain sensitivity alterations remain scarce. Within-subject designs offer greater statistical power and control for individual variability compared with prevalent cross-sectional approaches (12). Understanding whether CPM improvements produce generalized effects on pain sensitivity or modality-specific adaptations has important mechanistic and clinical implications. The neuroanatomical organization of nociceptive pathways, with distinct populations for thermal vs. mechanical pain (13), provides a mechanistic basis for potential modality specificity not systematically examined in CPM plasticity studies.

Descending pain modulatory systems originate from cortical and subcortical regions intimately involved in emotional processing, attention, and cognitive evaluation (14, 15). Consequently, psychological factors such as anxiety, depression, fear of pain, and treatment expectations may significantly influence CPM efficiency (16, 17).

Our prior work demonstrated that repeated CPM exposure enhanced efficiency in healthy adults, with high-exposure participants showing superior efficiency compared with low-exposure and no-exposure groups (*p* = 0.02, *η*^2^ = 0.05), with significant time-related improvements (*p* < 0.001, *η*^2^ = 0.23) (1), demonstrating CPM plasticity with repeated activation (1).

This planned secondary analysis extends our primary findings (1) by examining the distinct mechanistic question of whether individual differences in CPM plasticity are associated with concurrent changes in pain sensitivity, a question with different theoretical and clinical implications than the primary intervention effects.

The purpose of this planned secondary analysis was to determine (1) the extent to which changes in CPM efficiency were associated with alterations in pain sensitivity across QST modalities and (2) whether psychological factors demonstrated relationships with changes in CPM efficiency. We hypothesized that CPM efficiency improvements would be associated with reduced pain sensitivity across modalities and that psychological factors would demonstrate relationships with CPM changes.

## 2 Methods

### 2.1 Study design

The primary trial was approved by University of Florida IRB (IRB 202300187) and registered at ClinicalTrials.gov (NCT05783362). In brief, 60 people without pain were randomly assigned to one of three experimental arms: a high exposure (HE)/repeated training group, a low exposure/single session group (LE), and a true no exposure (NE)/no training control group. All participants completed demographic and psychological questionnaires and a battery of quantitative sensory tests to establish pain sensitivity. HE (*n* = 20) completed five sessions comprising four intervention sessions with baseline and final assessment including complete psychological questionnaire and QST procedures, scheduled every 48–72 h over 2 weeks. LE (*n* = 20) completed two sessions including single intervention with baseline and final assessment incorporating identical outcome measures, separated by 2 weeks. NE (*n* = 20) received no experimental CPM activation, as baseline assessment itself could represent “exposure” to the intervention. The participants attended two sessions with questionnaires and one QST session administered at the terminal session following a two-week interval. Importantly, the NE group did not undergo baseline QST assessment, which informs the analytical approach for baseline correlations described below.

Complete methodological details, including study flow diagrams and CONSORT information, have been reported previously (1).

### 2.2 Outcome measures

#### 2.2.1 Psychological measures

The Brief Pain Inventory (BPI) was administered but excluded from secondary analyses because of floor effects in this pain-free sample, where the majority of participants reported scores of 0 or near-0 on pain intensity and interference subscales.

##### 2.2.1.1 Depression

The Center for Epidemiological Studies Depression Scale (CES-D) assessed depressive symptoms across 20 items with total scores ranging 0–60, where higher scores indicate greater symptom severity (Cronbach *α*: 0.87) (18–20).

##### 2.2.1.2 Affective states

The Positive and Negative Affect Schedule (PANAS) quantified affective valence through 20 items, each scored on a 5-point Likert scale (range 10–50 per subscale), capturing both positive and negative affective dimensions (Cronbach *α* = 0.91 for the PANAS-P and 0.87 for the PANAS-N) (21).

##### 2.2.1.3 Anxiety

The Generalized Anxiety Disorder-7 (GAD-7) measured anxiety symptom severity using seven items rated on a 4-point scale from 0 (“not at all”) to 3 (“nearly every day”), yielding total scores of 0–21 (Cronbach *α* = 0.895) (22, 23).

##### 2.2.1.4 Fear of pain

The Fear of Pain Questionnaire-9 (FPQ-9) evaluated pain-related fear cognitions through nine items rated on a 5-point scale from 1 (“not at all”) to 5 (“extreme”), producing total scores ranging from 5 to 45 (Cronbach *α* = 0.873) (24, 25).

##### 2.2.1.5 Expectations

Pain-related expectations of pressure pain intensity were assessed using a 0–100 NRS scale anchored at zero being “No pressure pain expected at all” and 100 being “Most intense pressure pain expected.” This aligns with the NRS framework employed for reporting actual pain sensations across the investigation. Using an identical scale for both anticipated and perceived pain intensity preserves methodological uniformity.

##### 2.2.1.6 Anxiety about pain testing

A single-item 0–100 NRS scale for anxiety about pain testing was administered before every session of the protocol. The participants were instructed to provide a number ranging from 0 (“no anxiety about pain testing”) to 100 (“worst anxiety about pain testing imaginable”).

#### 2.2.2 QST measures

Pain intensity across all QST procedures was measured using the 101-point numeric rating scale (0–100), with anchors of “no pain” and “most intense pain sensation imaginable” (ICC = 0.95) (26–29).

##### 2.2.2.1 CPM-outcome (assessment protocol)

The participants received a suprathreshold pressure pain stimulus (pain-40) to the dominant foot webspace. They verbally indicated “stop” when experiencing pain equivalent to 40 on a 0–100 scale. Pressure increased at 1 kg/s until pain intensity reached 40/100, performed twice and averaged (2). The participants then received a thermal conditioning stimulus of contact heat (46.5 °C for 60 s) applied to the left thenar eminence (30), with pain ratings provided during exposure. This thermal conditioning stimulus was selected based on established protocols demonstrating reliable CPM induction with heat stimuli. The participants could withdraw their hand if heat became unbearable. Following heat removal, pain-40 was reapplied to the foot webspace. CPM was computed as the mean pressure pain-40 threshold (kg) from the initial test minus subsequent test. Negative values represent efficient pain modulation (31). CPM constituted our principal metric of endogenous inhibitory function.

##### 2.2.2.2 Pressure pain threshold

Mechanical pain thresholds were determined using a calibrated pressure algometer (AlgoMed, Israel) with a standardized 1 cm^2^ contact area applied perpendicular to the dominant first dorsal interosseous muscle at a consistent rate of 1 kg/s (32). The participants provided verbal indication of the transition point from pressure sensation to pain, with duplicate measurements averaged for statistical analysis. Duplicate measurements were used consistent with established protocols demonstrating acceptable test–retest reliability (ICC >0.80) for thermal and pressure pain assessments (26, 33), while minimizing participant burden given the comprehensive nature of our multisession protocol.

##### 2.2.2.3 Thermal pain threshold and tolerance

Heat pain thresholds and tolerance levels were established using a computer-controlled thermode system (TSAII, Medoc Inc., Israel) applied to the volar aspect of the dominant forearm (2). Temperature increased from a 35 °C baseline at 1 °C per second to a maximum safety cutoff of 51 °C. The participants identified both the warmth-to-pain transition point (threshold) and the maximum tolerable intensity (tolerance), with duplicate trials averaged for analysis. For participants who reached the 51 °C safety cutoff before indicating the threshold or tolerance, this temperature was recorded as their value for that measure; however, such ceiling effects were rare in our sample and did not substantially impact the distribution of scores.

Critically, at both threshold and tolerance temperatures, the participants provided pain intensity ratings using the 0–100 NRS. This dual measurement approach captures both the temperature ( °C) required for pain detection/tolerance and the pain intensity rating experienced at that temperature. Heat threshold ratings represent the pain intensity at the warmth-to-pain transition, while heat tolerance ratings represent the pain intensity at the maximum tolerable temperature (the participants indicated when they could no longer tolerate increasing temperature, regardless of their current pain rating). This approach allows a dissociation of threshold/tolerance shifts from intensity encoding changes.

##### 2.2.2.4 Aftersensations

Following thermode removal, the participants verbally reported pain intensity ratings using the same 0–100 NRS at 15-s intervals for a total duration of 60 s to assess residual pain sensations (34). Aftersensation measurements served as a secondary indicator of endogenous pain inhibitory function and central sensitization processes (34).

### 2.3 CPM intervention (training protocol)

To minimize practice effects, distinct stimuli were administered to different anatomical locations for intervention vs. evaluation. The participants received a suprathreshold pressure pain stimulus (pain-40) to the dominant foot webspace. Pressure increased at 1 kg/s until pain intensity reached 40/100, performed twice and averaged (2). Subsequently, the participants received a conditioning stimulus via non-dominant hand immersion in circulating water at 6 °C (males) or 8 °C (females) for 60 s, based on sex differences in cold pain sensitivity (35, 36). The participants rated cold pain during each of four 60-s trials and could withdraw their hand if intolerable. When withdrawal occurred or ratings exceeded 50/100, water temperature was elevated for the next trial; if ratings fell below 20, temperature was reduced up to 4 °C. This personalized protocol maintained comparable pain intensity across participants. Following each 60-s immersion, the participants withdrew their hand for 30 s, while pain-40 was reapplied to the foot webspace. The participants completed four immersion cycles. CPM was computed as the mean pressure pain-40 threshold (kg) from the initial test minus the subsequent test. Negative values signified efficient pain modulation (31).

### 2.4 Experimental procedures

Consenting participants were scheduled for individual testing blocks (90 min). Data were collected by the principal investigator, coinvestigator, and/or research assistant with direct supervision from the principal or coinvestigator. While research personnel were not blinded to group assignments, the participants remained blinded to their group allocation.

Psychological assessments were conducted at initial and final visits. Each session included expectation and anxiety questionnaires, followed by blood pressure measurement; participants exceeding 140/90 mmHg were excluded from that session. Subsequently, baseline QST (slow ramp, aftersensations, pressure pain threshold, and CPM-outcome) was performed. Following a 15-min rest period to permit normalization of pain sensitivity (37), HE and LE participants underwent the CPM-intervention protocol.

## 3 Statistical analysis

Power calculations using G*Power 3.1.9.7 based on an anticipated medium effect size (*f*^2^ = 0.15) for multiple regression determined 15 participants per group who provided >80% power (*α* = 0.05, two-tailed) for detecting associations between CPM efficiency changes and outcome measures (38). Enrollment was set at 20 per group to account for attrition and accommodate potentially smaller effect sizes.

Data analysis used R software, screening for outliers (>3 SD). Normality was examined through histograms, z-ratio skewness/kurtosis, the Shapiro–Wilk test, and the Kolmogorov–Smirnov test. Data meeting ≥3 of 4 criteria were considered normal. All regression variables met normality, except heat tolerance rating changes (*z* = −2.3); sensitivity analyses using log transformation yielded identical results, and therefore, untransformed values were reported.

Descriptive statistics were generated for demographics, psychological variables, and medical background. One-way ANOVA evaluated continuous measures; chi-square tests evaluated categorical measures across conditions. The chi-square tests verified equivalent sex representation [*χ*^2^(2) = 0.87, *p* = 0.65].

CPM calculation followed recommendations (8) using the difference between the pre-CPM minus post-CPM pressure pain-40 threshold. The CPM training protocol, which involved applying pressure pain as the test stimulus and cold water as the conditioning stimulus, served exclusively for training and was excluded from outcome measurements.

This planned secondary analysis examined associations between changes in CPM efficiency and pain sensitivity measures, as well as psychological factors, using multiple linear regression.

For Research Question 1, change in CPM efficiency served as a predictor in linear regression modeling with changes in pain sensitivity measures as separate outcomes, controlling for intervention arm and age. The intervention arm was included as a covariate to account for potential confounding from differences in exposure intensity between HE and LE groups, following recommendations for analyzing within-subject changes when the participants received different intervention intensities (12). The test of group differences in CPM efficiency using two-way ANOVA with the intervention arm (HE and LE) and time (preintervention, postintervention) as factors revealed no significant interaction between arm and time [*F*(1,76) = 0.73, *p* = 0.39, *η*^2^ = 0.006]. The absence of interaction supported the including arm as a covariate in subsequent analyses.

Baseline correlations between QST and psychological measures included only HE and LE groups (*n* = 40), as the NE group did not undergo baseline QST to avoid unintended CPM exposure from the assessment procedure itself. Final visit correlational analyses examining relationships between CPM efficiency and QST outcomes included all three groups (*n* = 60).

Our primary analyses focused on thermal pain measures, given the thermal nature of the conditioning stimuli used in both CPM protocols. Analyses involving pressure pain measures, aftersensations, and psychological factors were more exploratory, examining the potential generalization of CPM-related effects across modalities and domains.

For Research Question 2, change in CPM efficiency predicted changes in psychological factors (depression, anxiety, fear of pain, positive affect, negative affect, pain-related expectations, and anxiety about testing), with the intervention arm and age as covariates. Model assumptions were verified through residual analysis.

The NE group functioned as post-test-only control addressing natural history effects and testing familiarity. Postintervention correlational analyses examined relationships between final CPM efficiency and QST outcomes across all arms using Pearson or Spearman correlations.

Bonferroni correction addressed Type I error. For research question 1: *α* = 0.025 for pressure measures (two comparisons) and *α* = 0.01 for thermal measures (five comparisons). For research question 2: *α* = 0.007 for psychological measures (seven comparisons). Statistical significance was determined based on these adjusted alpha levels.

## 4 Results

### 4.1 Participants

As previously reported (1), 60 pain-free participants completed the study protocol (mean age = 37.0 ± 12.9 years, range 18–75 years; 51.7% female). Groups were well-matched on demographic and baseline psychological measures, with no significant between-group differences (all *p* > 0.05) and equivalent sex representation across groups [*χ*^2^(2) = 0.87, *p* = 0.65]. Complete demographic characteristics, CONSORT flow diagram, and study flow are available in (1). No intervention-related adverse events occurred. Descriptive statistics for QST measures are presented in Table 1.

**Table 1:** Descriptive statistics for QST measures by group and time point.

	High exposure (*n* = 20)		Low exposure (*n* = 20)	
Measure	Baseline	Final visit	Baseline	Final visit
CPM efficiency (kg)	0.43 (0.76)	1.29 (0.72)	0.21 (0.66)	0.83 (0.47)
Heat threshold ( °C)	43.29 (4.14)	43.58 (3.12)	44.31 (2.27)	43.7 (2.62)
Heat threshold rating (0–100)	26.62 (14.49)	18.38 (13.06)	31.45 (12.02)	33.08 (17.86)
Heat tolerance ( °C)	47.92 (2.22)	48.8 (1.41)	48.54 (1.2)	48.36 (1.19)
Heat tolerance rating (0–100)	74.25 (15.75)	69.62 (21.56)	74.92 (16.66)	73 (17.31)
Aftersensations (0–100)	−8.5 (7.85)	−2.25 (3.18)	−8.4 (10.71)	−9.8 (11.56)
Pressure pain threshold (kg)	4.17 (1.8)	4.37 (1.34)	4.51 (2.03)	3.76 (1.33)
Pressure pain threshold rating (0–100)	26.48 (15.23)	22.42 (12.77)	32.73 (17.43)	32.05 (21.17)

Values presented as mean (SD).

CPM, conditioned pain modulation; QST, quantitative sensory testing.

### 4.2 Association between changes in CPM efficiency and quantitative sensory testing measures (HE and LE)

#### 4.2.1 Thermal pain measures

##### 4.2.1.1 Heat threshold temperature

**Figure 1:**
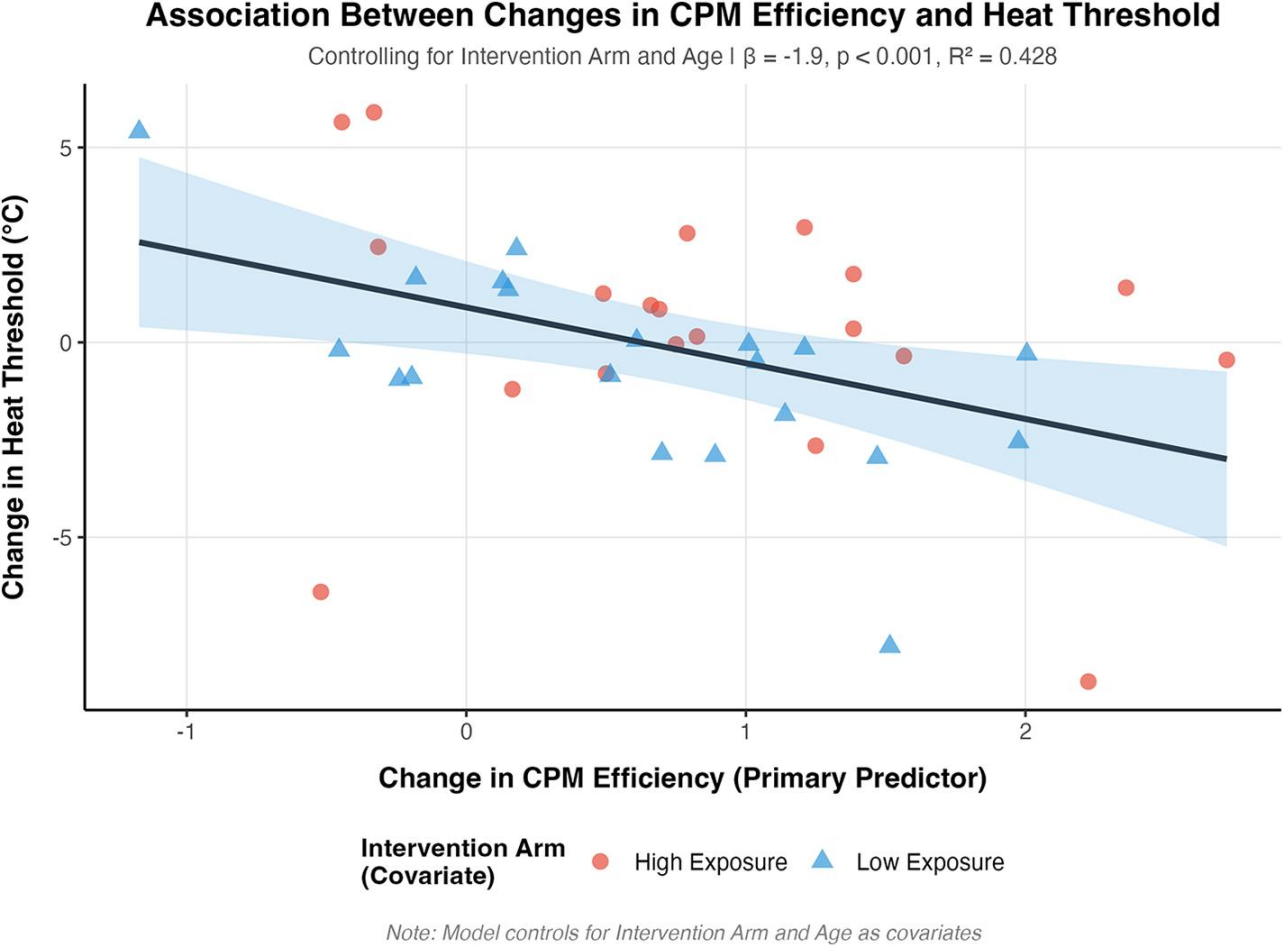
Relationship between change in conditioned pain modulation (CPM) efficiency and change in heat threshold temperature from baseline to postintervention. Data points represent individual participants from high exposure (triangles, *n* = 20) and low exposure (circles, *n* = 20) groups. The solid line represents the linear regression fit (*β* = −1.90, SE = 0.45, *p* < 0.001), and the shaded region indicates the 95% confidence interval. Model statistics: *F*(3,36) = 8.97, *p* < 0.001, *R*^2^ = 0.43, adjusted *R*^2^ = 0.38. More negative CPM efficiency change scores indicate improved pain modulation; positive heat threshold changes indicate increased temperature required for pain detection.

The regression model examining changes in the heat threshold was statistically significant [*F*(3,36) = 8.97, *p* < 0.001, *R*^2^ = 0.43, adjusted *R*^2^ = 0.38] as shown in Figure 1. Change in CPM efficiency demonstrated a significant association with heat threshold changes (*β* = −1.90, SE = 0.45, *p* < 0.001), indicating that greater improvements in CPM efficiency (more negative values) were associated with increased heat thresholds. Age demonstrated a significant negative association with heat threshold changes (*β* = −0.11, SE = 0.03, *p* < 0.001), consistent with established age-related changes in thermal pain processing. The intervention arm effect approached significance (*β* = −1.40, SE = 0.77, *p* = 0.076).

##### 4.2.1.2 Heat tolerance temperature

**Figure 2:**
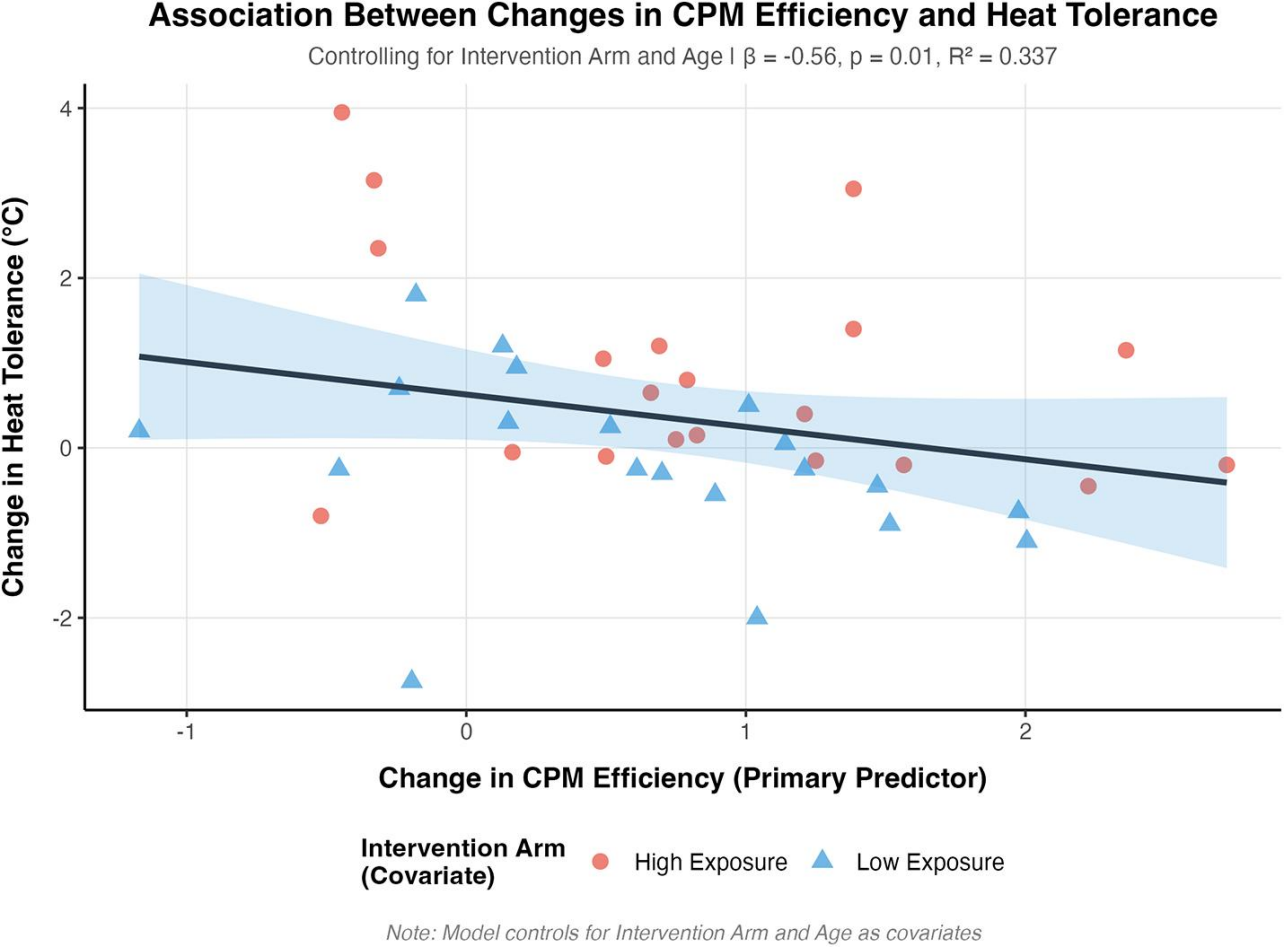
Relationship between change in conditioned pain modulation (CPM) efficiency and change in heat tolerance temperature from baseline to postintervention. Data points represent individual participants from high exposure (triangles, *n* = 20) and low exposure (circles, *n* = 20) groups. The solid line represents the linear regression fit (*β* = −0.56, SE = 0.21, *p* = 0.010), and the shaded region indicates the 95% confidence interval. Model statistics: *F*(3,36) = 6.11, *p* = 0.002, *R*^2^ = 0.34, adjusted *R*^2^ = 0.28. More negative CPM efficiency change scores indicate improved pain modulation; positive heat tolerance changes indicate increased maximum tolerable temperature.

The regression model for heat tolerance changes was statistically significant [*F*(3,36) = 6.11, *p* = 0.002, *R*^2^ = 0.34, adjusted *R*^2^ = 0.28] as shown in Figure 2. Change in CPM efficiency showed a significant association with heat tolerance changes (*β* = −0.56, SE = 0.21, *p* = 0.010). The intervention arm demonstrated a significant effect (*β* = −1.20, SE = 0.35, *p* = 0.002), while age showed a trend toward significance (*β* = −0.03, SE = 0.01, *p* = 0.073).

##### 4.2.1.3 Heat threshold rating

The regression model was not significant [*F*(3,36) = 2.27, *p* = 0.097, *R*^2^ = 0.16]. No significant associations were observed for CPM efficiency change (*p* = 0.23), intervention arm (*p* = 0.061), or age (*p* = 0.57) (Supplementary Table S1).

##### 4.2.1.4 Heat tolerance rating

The regression model was not significant [*F*(3,36) = 0.25, *p* = 0.86, *R*^2^ = 0.02]. No significant associations were observed for CPM efficiency change (*p* = 0.58), intervention arm (*p* = 0.59), or age (*p* = 0.75) (Supplementary Table S1).

##### 4.2.1.5 Aftersensations

The regression model was not significant [*F*(3,36) = 1.61, *p* = 0.20, *R*^2^ = 0.12]. CPM efficiency change showed no significant association (*p* = 0.80). The intervention arm demonstrated an effect (*p* = 0.041) that did not meet the Bonferroni-corrected threshold. Age showed no association (*p* = 0.62) (Supplementary Table S1).

#### 4.2.2 Pressure pain measures

##### 4.2.2.1 Pressure pain threshold

The regression model was not significant [*F*(3,36) = 1.11, *p* = 0.36, *R*^2^ = 0.08]. No significant associations were observed for CPM efficiency change (*p* = 0.69), intervention arm (*p* = 0.080), or age (*p* = 0.77) (Supplementary Table S1).

##### 4.2.2.2 Pressure pain threshold rating

The regression model was not significant [*F*(3,36) = 0.32, *p* = 0.81, *R*^2^ = 0.03]. No significant associations were observed for CPM efficiency change (*p* = 0.62), intervention arm (*p* = 0.51), or age (*p* = 0.65) (Supplementary Table S1).

### 4.3 Baseline correlations between pain sensitivity and psychological measures (HE and LE)

Baseline correlations included only HE and LE groups (*n* = 40) because the NE group did not undergo baseline QST assessment. Baseline correlations (*n* = 40) revealed selective associations. Fear of pain negatively correlated with the heat threshold (*r* = −0.62, *p* < 0.001), heat tolerance (*r* = −0.50, *p* = 0.001), and pressure pain threshold (*r* = −0.44, *p* = 0.004). Anxiety negatively correlated with the pressure pain threshold (*r* = −0.33, *p* = 0.04). Associations with depression, positive affect, and negative affect were non-significant (all *p* > 0.07), consistent with restricted range in this healthy sample. Complete statistics are provided in Supplementary Table S5.

### 4.4 Association between change in CPM efficiency and psychological factors (HE and LE)

No significant associations were observed between changes in CPM efficiency and any psychological measures, including depression (*p* = 0.535), anxiety (*p* = 0.218), negative affect (*p* = 0.912), positive affect (*p* = 0.416), fear of pain (*p* = 0.064), change in expectations (*p* = 0.073), or anxiety about pain testing (*p* = 0.840). Complete regression statistics are presented in Supplementary Table S2.

### 4.5 Association between CPM efficiency and quantitative sensory testing measures at final visit (HE, LE, and NE)

#### 4.5.1 Thermal pain measures

CPM efficiency at final visit showed no significant associations with the heat threshold (*p* = 0.819), heat threshold rating (*p* = 0.886), heat tolerance (*p* = 0.397), or heat tolerance rating (*p* = 0.417). Complete regression results are provided in Supplementary Table S3.

#### 4.5.2 Pressure pain measures

CPM efficiency showed no significant association with the pressure pain threshold (*p* = 0.336) or pressure pain threshold rating (*p* = 0.092). The intervention arm demonstrated a significant effect on the pressure pain threshold rating (*β* = 7.61, SE = 2.86, *p* = 0.010). Complete statistics are presented in Supplementary Table S3.

### 4.6 Association between CPM efficiency and psychological measures at final visit (HE, LE, and NE)

No significant associations were observed between final visit CPM efficiency and depression (*p* = 0.934), anxiety (*p* = 0.870), negative affect (*p* = 0.900), positive affect (*p* = 0.302), fear of pain (*p* = 0.193), anxiety about pain testing (*p* = 0.582), or expectations (*p* = 0.416). Full regression results are available in Supplementary Table S4.

## 5 Discussion

This study revealed a selective modality-specific neuroplasticity in endogenous pain modulation: improvements in CPM efficiency were significantly associated with increases in the thermal pain threshold and thermal pain tolerance temperatures but not with pain intensity ratings at these thresholds, pressure pain sensitivity, aftersensations or psychological factors. These findings have important implications for understanding pain processing mechanisms and clinical pain assessment.

The longitudinal design examining within-subject changes rather than cross-sectional between-subject comparisons represents a key methodological advance. The dissociation between the thermal pain threshold and thermal pain tolerance and pain intensity ratings provides novel mechanistic insights into the specificity of CPM-induced neuroplastic adaptations. Our longitudinal approach reveals associations between CPM plasticity and pain sensitivity changes that were not apparent in cross-sectional analyses at the study endpoint, suggesting that within-subject tracking of CPM changes may offer greater sensitivity for understanding endogenous pain modulation dynamics.

### 5.1 Primary findings: selective thermal threshold and tolerance associations

Improvements in CPM efficiency predicted increases in both heat threshold temperature and heat tolerance temperature with large effect sizes. Participants who demonstrated greater enhancement in endogenous inhibitory capacity showed elevated thermal pain thresholds, requiring higher temperatures to perceive pain and tolerating higher maximum temperatures.

These findings support the hypothesis that repeated activation of descending inhibitory pathways through CPM protocols produces neuroplastic adaptations that enhance thermal pain processing at the detection level. This aligns with evidence that CPM mechanisms involve supraspinal descending modulation of spinal nociceptive processing (2, 33). The overlapping neural substrates between CPM and thermal pain processing, including spinothalamic tract pathways and RVM descending projections, likely explain why CPM efficiency improvements specifically influenced thermal rather than mechanical pain detection (9, 10).

### 5.2 Changes in threshold and tolerance temperatures without changes in pain intensity ratings: mechanistic insights

The most mechanistically informative finding was that the participants required higher temperatures to report pain onset (threshold) and to reach maximum tolerance, but once these higher temperatures were reached, they rated the pain similarly to baseline. This pattern suggests that CPM-induced plasticity enhanced the *thermal nociceptive detection system* without altering the encoding of pain intensity once suprathreshold stimulation occurred.

This finding aligns with the function of descending inhibition from the PAG and RVM to the spinal dorsal horn, which primarily modulates nociceptive transmission at the initial detection level (9, 10).

The mechanism for this modality-specific effect lies in the organization of both the RVM and the spinal dorsal horn. The RVM contains heterogeneous neuronal populations that can demonstrate modality-specific responses (9). Furthermore, the spinal dorsal horn exhibits laminar organization wherein thermal nociception (primarily processed in superficial laminae I-II via C-fibers and Aδ-fibers) and mechanical nociception (processed in deeper laminae via Aβ-fibers) occupy distinct anatomical territories. Descending projections from the RVM can therefore selectively target thermally responsive neurons in superficial laminae, while having less influence on mechanoreceptive processing in deeper laminae (13).

The absence of aftersensation changes further supports this spinal-level threshold adaptation hypothesis. Aftersensations reflect inhibition occurring after temporal summation and central sensitization processes involving dorsal horn wind-up and cortical-limbic amplification (34). The lack of aftersensation changes suggests that CPM-induced plasticity occurred at the initial detection level rather than affecting central amplification mechanisms (39).

### 5.3 Why thermal but not mechanical modality? Pathway-specific plasticity

The thermal specificity reflects pathway-specific neuroplasticity in descending modulation. Thermal and mechanical nociception are transmitted via distinct afferent populations and spinal pathways: thermal pain via C-fibers and Aδ-fibers through the spinothalamic tract (primarily superficial dorsal horn laminae), vs. mechanical pain via Aβ-fibers and dorsal column pathways (deeper laminae) (13). Repeated CPM activation may have selectively strengthened descending inhibitory projections onto thermal-responsive dorsal horn neurons in superficial laminae, while sparing mechanoreceptive pathways in deeper laminae (32). A critical contributing factor is the thermal conditioning stimulus overlap in both protocols: the CPM assessment employed heat (46.5 °C contact thermode) as the conditioning stimulus, while the CPM intervention utilized cold water immersion (6 °C–8 °C). Both conditioning stimuli activate thermosensitive nociceptive pathways, which may have induced pathway-specific adaptations through Hebbian-like mechanisms wherein repeated coactivation of thermal nociceptive circuits strengthened their inhibitory modulation (40). This thermal modality consistency across protocols may explain why improvements transferred specifically to thermal pain outcomes. In addition, thermal pathways exhibit greater short-term neuroplastic capacity compared with mechanical pathways, as reflected in their greater variability and context-dependence (2).

### 5.4 Age effects: independent constraint on plasticity magnitude

Age demonstrated a significant negative association with heat threshold changes (*β* = −0.11, SE = 0.03, *p* < 0.001), with older participants showing smaller improvements regardless of CPM efficiency changes. This aligns with evidence documenting age-related declines in endogenous pain modulation (41), likely reflecting reduced descending inhibitory function or decreased opioidergic receptor density (42). However, a moderation analysis revealed no significant age×CPM efficiency interaction (*β* = −0.016, SE = 0.029, *p* = 0.598), indicating similar mechanisms across ages. This suggests that interventions targeting endogenous inhibition may benefit older adults through identical mechanisms but require more intensive protocols to achieve comparable improvements.

### 5.5 Psychological factors: trait stability

The lack of significant associations between CPM efficiency changes and psychological factors (depression, anxiety, fear of pain, affect, and expectations) likely reflects temporal mismatch: psychological measures assessed stable trait-like constructs unlikely to change substantially over 2 weeks, whereas CPM efficiency demonstrated state-like plasticity with repeated exposure. Our primary analysis confirmed this: GAD-7, affect measures, CES-D, expectations, and FPQ showed no significant intervention or time effects (1). Although baseline cross-sectional analyses revealed significant correlations between fear of pain and thermal thresholds (*r* = −0.62, *p* < 0.001) and anxiety with the pressure pain threshold (*r* = −0.33, *p* = 0.04), these between-subject associations did not translate into within-subject change associations.

### 5.6 Cross-sectional vs. longitudinal findings: methodological implications

A striking finding was the absence of significant cross-sectional associations between CPM efficiency and pain measures at final visit, despite robust longitudinal associations between CPM changes and thermal threshold changes. This pattern underscores that within-subject changes provide a more sensitive detection of CPM-pain sensitivity relationships than between-subject comparisons (12). Within-subject change scores naturally control for individual differences in genetic polymorphisms, skin thickness, baseline descending modulation, and psychological states (2, 43), isolating intervention-related plasticity.

### 5.7 Limitations

Several limitations warrant consideration. First, exclusive recruitment of pain-free adults limits generalizability to clinical pain populations where impaired baseline CPM and greater pain sensitivity may reveal different patterns. Second, the 2-week intervention period may have been sufficient for spinal-level thermal threshold adaptations but insufficient for broader neuroplastic changes affecting pain ratings, pressure sensitivity, or psychological factors. Third, without neuroimaging or neurochemical biomarkers, proposed mechanisms (spinal-level plasticity, pathway-specific modulation) remain speculative pending direct neurobiological evidence. We note that while our primary confirmatory analyses focused on thermal pain outcomes (given the thermal conditioning stimuli used), the null findings for pressure pain and psychological measures were exploratory and should be interpreted cautiously.

Future studies in clinical pain populations with impaired baseline CPM may reveal relationships obscured in healthy samples. Neuroimaging studies could elucidate neural mechanisms underlying the selective CPM-thermal pain threshold association, identifying specific brainstem and spinal circuits responsible for modality-specific pain modulation.

## 6 Conclusion

CPM protocols employing thermal conditioning stimuli function as thermal-specific biomarkers of nociceptive detection rather than global pain sensitivity measures. CPM efficiency improvements predicted increased heat threshold and tolerance temperatures without corresponding pain intensity rating changes, demonstrating that repeated thermal CPM exposure enhances thermal nociceptive detection through descending modulation. The thermal-specific nature of these findings, with effects on thermal but not mechanical pain measures, may reflect the shared thermal modality between the conditioning stimulus (heat for assessment and cold water for intervention) and the thermal pain outcomes. For clinical application, assessment protocols should incorporate both detection thresholds and suprathreshold ratings to capture treatment effects comprehensively, and researchers should consider modality specificity when designing CPM interventions.

## Data Availability

The raw data supporting the conclusions of this article will be made available by the authors, without undue reservation.
