# Supplementary material for: Association between changes in conditioned pain modulation efficiency and pain sensitivity: a randomized controlled trial
Source: Front Pain Res (Lausanne). 2026 Mar 26;7:1802533. doi: 10.3389/fpain.2026.1802533 (PMC13062177; doi:10.3389/fpain.2026.1802533)
Supplement: Supplementary file 1 [file Datasheet1.pdf]

Supplementary Table S1.

Multiple Regression Analysis: Association Between Changes in CPM Efficiency and Thermal Pain Measures (HE and LE Groups)

Outcome	Predictor	β	SE	t	p	95% CI	R^2	Adjusted R^2	F	p
Heat Threshold Rating	Change in CPM	-3.38	2.76	-1.23	0.228	[-8.94, 2.19]	0.159	0.089	2.27	0.097
	Arm	9.03	4.67	1.93	0.061	[-0.38, 18.44]				
	Age	0.10	0.18	0.57	0.572	[-0.26, 0.47]				
Heat Tolerance Rating	Change in CPM	1.88	3.39	0.55	0.583	[-4.96, 8.72]	0.020	-0.062	0.25	0.863
	Arm	3.17	5.75	0.55	0.585	[-8.43, 14.77]				
	Age	-0.07	0.22	-0.32	0.748	[-0.52, 0.38]				
Aftersensations	Change in CPM	-0.55	2.16	-0.26	0.800	[-4.91, 3.81]	0.119	0.045	1.61	0.203
	Arm	-7.78	3.67	-2.12	0.041 †	[-15.18, -0.38]				
	Age	0.07	0.14	0.49	0.624	[-0.21, 0.35]				
Pressure Pain Threshold (UE)	Change in CPM	-0.13	0.32	-0.40	0.691	[-0.77, 0.51]	0.084	0.008	1.11	0.360
	Arm	-0.98	0.54	-1.80	0.080	[-2.08, 0.12]				
	Age	-0.01	0.02	-0.30	0.768	[-0.05, 0.04]				
Pressure Pain Rating (UE)	Change in CPM	1.66	3.32	0.50	0.620	[-5.04, 8.36]	0.026	-0.056	0.32	0.813
	Arm	3.79	5.63	0.67	0.505	[-7.56, 15.14]				
	Age	-0.10	0.22	-0.45	0.654	[-0.54, 0.34]				

df = 3, 36 for all models. Bonferroni-corrected α thresholds: 0.01 for thermal measures (5 comparisons), 0.025 for pressure measures (2 comparisons). Does not meet Bonferroni-corrected threshold.

Supplementary Table S2.

Multiple Regression Analysis: Association Between Changes in CPM Efficiency and Psychological Measures (HE and LE Groups)

Outcome	Predictor	β	SE	t	p	95% CI	R^2	Adjusted R^2	F	p
Depression (CESD)	Change in CPM	0.70	1.12	0.63	0.535	[-1.56, 2.97]	0.019	-0.063	0.23	0.875
	Arm	1.13	1.90	0.59	0.556	[-2.72, 4.98]				
	Age	0.02	0.07	0.33	0.740	[-0.12, 0.17]				
Anxiety (GAD-7)	Change in CPM	-0.77	0.62	-1.25	0.218	[-2.02, 0.47]	0.069	-0.008	0.89	0.455
	Arm	0.35	1.05	0.33	0.740	[-1.76, 2.46]				
	Age	-0.05	0.04	-1.16	0.254	[-0.13, 0.04]				
Negative Affect	Change in CPM	0.10	0.88	0.11	0.912	[-1.68, 1.87]	0.003	-0.080	0.04	0.991
	Arm	-0.08	1.49	-0.05	0.959	[-3.09, 2.94]				
	Age	-0.02	0.06	-0.27	0.791	[-0.14, 0.10]				
Positive Affect	Change in CPM	0.89	1.08	0.82	0.416	[-1.30, 3.08]	0.021	-0.061	0.26	0.857
	Arm	-0.32	1.84	-0.18	0.861	[-4.03, 3.38]				
	Age	0.01	0.07	0.19	0.848	[-0.13, 0.16]				
Fear of Pain (FPQ)	Change in CPM	2.14	1.12	1.91	0.064†	[-0.12, 4.40]	0.119	0.045	1.62	0.202
	Arm	-0.81	1.89	-0.43	0.671	[-4.64, 3.01]				
	Age	-0.03	0.07	-0.38	0.706	[-0.18, 0.12]				
Change in Expectations	Change in CPM	2.31	1.25	1.85	0.073†	[-0.22, 4.84]	0.105	0.031	1.41	0.255
	Arm	1.63	2.12	0.77	0.447	[-2.66, 5.93]				
	Age	-0.03	0.08	-0.33	0.743	[-0.19, 0.14]				
Anxiety about Testing (VAS)	Change in CPM	1.18	5.83	0.20	0.840	[-10.60, 12.97]	0.033	-0.047	0.41	0.744
	Arm	9.88	9.89	1.00	0.324	[-10.06, 29.82]				
	Age	-0.16	0.38	-0.43	0.671	[-0.93, 0.61]				

Note. df = 3, 36 for all models. Bonferroni-corrected α threshold: 0.0083 for psychological measures (7 comparisons). † Does not meet Bonferroni-corrected threshold.

Supplementary Table S3.

Multiple Regression Analysis: Association Between Final Visit CPM Efficiency and Thermal Pain Measures (HE, LE and NE Groups)

Outcome	Predictor	β	SE	t	p	95% CI	R^2	Adjusted R^2	F	p
Heat Threshold	CPM Efficiency	-0.13	0.59	-0.23	0.819	[-1.31, 1.04]	0.018	-0.035	0.34	0.795
	Arm	-0.35	0.48	-0.73	0.470	[-1.30, 0.61]				
	Age	0.02	0.03	0.61	0.543	[-0.04, 0.08]				

Heat Threshold Rating	CPM Efficiency	-0.48	3.35	-0.14	0.886	[-7.22, 6.25]	0.124	0.077	2.65	0.058
	Arm	6.31	2.72	2.32	0.024	[0.84, 11.79]				
	Age	0.26	0.17	1.58	0.119	[-0.07, 0.60]				
Heat Tolerance	CPM Efficiency	0.24	0.28	0.85	0.397	[-0.32, 0.79]	0.116	0.068	2.44	0.073
	Arm	-0.29	0.23	-1.28	0.206	[-0.74, 0.16]				
	Age	0.03	0.01	1.92	0.060	[-0.001, 0.05]				
Heat Tolerance Rating	CPM Efficiency	3.08	3.76	0.82	0.417	[-4.48, 10.63]	0.033	-0.019	0.64	0.595
	Arm	3.59	3.05	1.18	0.244	[-2.55, 9.73]				
	Age	-0.07	0.19	-0.35	0.729	[-0.45, 0.32]				
Pressure Pain Threshold (UE)	CPM Efficiency	0.31	0.32	0.97	0.336	[-0.33, 0.95]	0.023	-0.030	0.43	0.732
	Arm	0.03	0.26	0.10	0.918	[-0.49, 0.54]				
	Age	0.01	0.02	0.62	0.535	[-0.02, 0.04]				
Pressure Pain Threshold (UE) Rating	CPM Efficiency	6.06	3.53	1.72	0.092	[-1.04, 13.15]	0.133	0.086	2.85	0.045
	Arm	7.61	2.86	2.66	0.010*	[1.86, 13.36]				
	Age	-0.06	0.18	-0.33	0.746	[-0.42, 0.30]				

Note. df = 3, 56 for all models. Bonferroni-corrected α thresholds: 0.01 for thermal measures, 0.025 for pressure measures. *Meets Bonferroni-corrected threshold. † Does not meet Bonferroni-corrected threshold.

Supplementary Table S4.

Multiple Regression Analysis: Association Between Final Visit CPM Efficiency and Psychological Measures (HE, LE and NE Groups)

Outcome	Predictor	β	SE	t	p	95% CI	R^2	Adjusted R^2	F	p
Depression (CESD)	CPM Efficiency	0.17	2.06	0.08	0.934	[-3.97, 4.32]	0.047	-0.004	0.92	0.437
	Arm	1.06	1.67	0.63	0.530	[-2.30, 4.41]				
	Age	-0.15	0.10	-1.45	0.152	[-0.36, 0.06]				
Anxiety (GAD-7)	CPM Efficiency	0.13	0.81	0.16	0.870	[-1.48, 1.75]	0.002	-0.052	0.03	0.992
	Arm	-0.01	0.65	-0.02	0.987	[-1.32, 1.30]				
	Age	-0.01	0.04	-0.24	0.808	[-0.09, 0.07]				
Negative Affect	CPM Efficiency	0.18	1.40	0.13	0.900	[-2.63, 2.99]	0.023	-0.029	0.44	0.725
	Arm	0.27	1.14	0.24	0.812	[-2.01, 2.55]				
	Age	-0.08	0.07	-1.08	0.284	[-0.22, 0.07]				
Positive Affect	CPM Efficiency	1.70	1.63	1.04	0.302	[-1.58, 4.98]	0.020	-0.033	0.37	0.772

	Arm	0.50	1.33	0.38	0.709	[-2.16, 3.16]				
	Age	-0.01	0.08	-0.07	0.942	[-0.17, 0.16]				
Fear of Pain (FPQ)	CPM Efficiency	1.97	1.50	1.32	0.193	[-1.04, 4.98]	0.050	-0.001	0.97	0.412
	Arm	0.28	1.21	0.23	0.819	[-2.16, 2.72]				
	Age	-0.07	0.07	-0.97	0.335	[-0.22, 0.08]				
Anxiety about Testing (VAS)	CPM Efficiency	2.52	4.54	0.55	0.582	[-6.60, 11.64]	0.034	-0.018	0.66	0.581
	Arm	4.28	3.69	1.16	0.251	[-3.13, 11.69]				
	Age	-0.14	0.23	-0.60	0.551	[-0.60, 0.32]				
Expectations	CPM Efficiency	-0.90	1.10	-0.82	0.416	[-3.12, 1.31]	0.015	-0.038	0.28	0.842
	Arm	-0.54	0.89	-0.61	0.545	[-2.33, 1.25]				
	Age	-0.01	0.06	-0.19	0.850	[-0.12, 0.10]				

Note: df = 3, 56 for all models. Bonferroni-corrected α: 0.0083 for psychological measures (7 comparisons). No associations met corrected significance threshold.

Supplementary Table S5.

Baseline Correlations Between Pain Sensitivity Measures and Psychological Factors

Note: Values shown are Pearson correlation coefficients with p-values in parentheses. Bold values indicate statistically significant correlations (p < 0.05).

Pain Sensitivity Measure	Depression (CES-D)	Anxiety (GAD-7)	Fear of Pain (FPQ-9)	Positive Affect (PANAS-P)	Negative Affect (PANAS-N)
Heat Threshold (°C)	−0.27 (p = 0.09)	−0.02 (p = 0.88)	**−0.62 (p < 0.001)**	0.20 (p = 0.22)	−0.25 (p = 0.12)
Heat Threshold Rating	−0.03 (p = 0.86)	−0.06 (p = 0.72)	−0.25 (p = 0.12)	−0.20 (p = 0.22)	−0.11 (p = 0.51)
Heat Tolerance (°C)	−0.24 (p = 0.14)	−0.17 (p = 0.31)	**−0.50 (p = 0.001)**	0.25 (p = 0.12)	−0.29 (p = 0.07)
Heat Tolerance Rating	0.13 (p = 0.42)	−0.08 (p = 0.63)	0.23 (p = 0.15)	0.00 (p = 0.98)	0.16 (p = 0.33)
Pressure Pain Threshold (kg)	−0.13 (p = 0.43)	**−0.33 (p = 0.04)**	**−0.44 (p = 0.004)**	0.29 (p = 0.07)	−0.25 (p = 0.12)
Pressure Pain Threshold Rating	0.23 (p = 0.16)	0.20 (p = 0.22)	0.12 (p = 0.45)	−0.22 (p = 0.18)	0.15 (p = 0.34)

Table Note: Fear of pain demonstrated consistent negative associations with pain detection thresholds and tolerance, indicating that individuals with higher fear of pain exhibited lower baseline pain thresholds. Despite these baseline cross-sectional relationships,

changes in CPM efficiency showed no associations with changes in psychological factors, suggesting CPM-induced neuroplasticity operates through mechanisms relatively independent of these cognitive-emotional influences.
